# Notes and News

**Published:** 1898-11-12

**Authors:** 


### NOTES AND NEWS.

(COLLECTED AND COMMUNICATED.)

THE annual meeting of the Governors of the Royal Hospital for Incurables will be held at Cannon Street Hotel on Friday, November 25th, at eleven o'clock.

THE buildings of St. Mary's Hospital, Manchester, so long delayed owing to difficulties arising out of the amalgamation scheme with the Manchester Southern Hospital, have now been commenced.

IT is proposed to rebuild the Belfast Maternity Hospital, and £6,000 is asked for the purpose. The present hospital, which has done a useful work for the past one hundred years, is now totally inadequate and unsuitable to modern requirements.

THE new Cottage Hospital at Thetford has been opened by Lady Iveagh. The institution is to serve as a Jubilee memorial, the scheme having originated with Mr. T. Bidwell, who gave the site and £400 towards the building.

THE new north wing at the Stockport Infirmary, the foundation-stone of which has just been laid by the Mayor, will consist of a pavilion block, four storeys high, in a line with the present buildings, and a laundry block and mortuary block at the rear of the institution; in the pavilion, two wards for fourteen beds in each, offices, servants' apartments, operating theatre, and two small surgical wards for two beds in each, matron's room, day and recreation rooms, various store rooms, and sanitary arrangements.

THE Committee of the Essex and Colchester Hospital have just secured the services of Major Becher as secretary. As it has been found necessary to employ the whole time of one person for the secretarial work of the hospital, Mr. Bland, who has done good service for years past, found it necessary to resign, as he has other duties. All those connected with the management of the hospital have shown themselves anxious to bring the institution into line with the best administered provincial hospitals, and this last step is in the right direction. The larger salary now paid for the entire services of an energetic man should be returned threefold in increased subscriptions and donations.

MR. B. EVANS has presented an operating theatre to the Swansea Hospital. Sir William MacCormac, in performing the opening ceremony, stated that the theatre was everything its best friends could require. It had no corners, no angles, and from floor to ceiling it was rounded off, and it had been supplied with all the equipments which were regarded as indispensable in a modern operating theatre.

WE congratulate the working men of Coventry on the endeavour they make to support their hospital. The working men's contributions to the Coventry and Warwickshire Hospital represent nearly twice the amount of ordinary annual subscriptions. Such a fact shows that a proper pride and a feeling of interest and responsibility is present amongst the working classes at Coventry which might well be widely emulated.

A CONSUMPTION Hospital has been opened for Chicago and the surrounding districts. Provision is made for 339 patients in a well-situated building. The structure is three storeys high. The third floor has wards in which treatment by compressed air can be carried out. One ward in the hospital is set apart for insane consumptive patients. The institution is attached to the workhouse at Dunning.

THE new Sanatorium or Infectious Diseases Hospital for the Borough of Brighton which has been opened at Brighton was commenced in 1896, to replace the temporary building in use until the present time. The new institution consists of administration block, discharge block, porter's lodge, new disinfecting station, and one ward pavilion. The buildings are substantially built with red kiln facing bricks, relieved by Portland stone sills, lintols, and copings. All the buildings are rendered as fireproof as possible. Internally, glazed brickwork and tiles have been largely used. The sanitary fittings have been most carefully chosen, and electric light has been installed throughout. Patients are now admitted free, except in the case of paupers and private cases. The reception of eighty patients is provided for, the accommodation being distributed over ten wards. The ward floors are of terrazzo, and the walls of opalite and Reith's cement, painted.

AT the forthcoming annual meeting of the Royal Hospital for Incurables Mrs. Georgina Hill intends to move a resolution, "That, in view of the fact that by far the larger proportion of the inmates of the Royal Hospital for Incurables are women, it is desirable that women should be added to the board of management." In support of her action, Mrs. Hill quotes the Earl of Aberdeen, who, when president of the Committee of Inquiry into the affairs of the hospital in 1893, wrote: "I am of opinion that, however valuable and beneficial the visits of ladies as at present to the inmates of the institution may be, it would be desirable that a certain number of ladies should be asked to join the committee or board of management. In this way the special experience of ladies would be made available, while there would not be the risk of any inconvenience or went of harmonious co-operation such as might, in the opinion of some people, exist, if a separate committee of ladies were formed."

